# Management of Peri‐Implant Soft Tissue Dehiscence Using Laterally Closed Tunneling With a Connective Tissue Graft: A Case Report

**DOI:** 10.1002/ccr3.73440

**Published:** 2026-08-31

**Authors:** Hisham Tarek

**Affiliations:** ^1^ Oral Medicine and Periodontology Department, Faculty of Dentistry The British University in Egypt Cairo Egypt

**Keywords:** case report, de epithelized free gingival grafts, dental implants, peri‐implant diseases, tunneling technique

## Abstract

Peri‐implant soft tissue dehiscence is an esthetic complication for which coronally advanced flap with connective tissue grafting remains a predictable treatment, whereas evidence for laterally closed tunneling (LCT) around implants is limited. This single‐patient case report describes a 4 × 3 mm labial peri‐implant soft tissue dehiscence at an immediately placed implant replacing the maxillary left central incisor. LCT combined with a de‐epithelialized free gingival graft and limited implantoplasty was performed. At 24 months, complete coverage was maintained; buccal soft tissue thickness increased from 1.0 to 2.7 mm, keratinized tissue width from 1 to 3 mm, Pink Esthetic Score from 6 to 12, Mucosal Scarring Index from 3 to 0, and patient satisfaction from 4 to 10. Standardized periapical radiographs showed stable interproximal crestal bone levels without evidence of progressive bone loss. In this single case, LCT with a de‐epithelialized free gingival graft was associated with stable soft tissue coverage and favorable esthetic and patient‐reported outcomes over 24 months; further studies are required to determine reproducibility and predictability.

## Key Clinical Message

Laterally closed tunneling combined with a de epithelized free gingival graft represents a minimally invasive approach for managing peri‐implant soft tissue dehiscence, achieving stable soft tissue coverage and enhanced esthetics while preserving papillary architecture in the esthetic zone.

## 1 Introduction

Long‐term implant success depends not only on osseointegration but also on stable, esthetically integrated peri‐implant soft tissues [1, 2]. Labial peri‐implant soft tissue deficiencies may expose implant components and compromise esthetics and patient satisfaction [3, 4].

Peri‐implant soft tissue dehiscence/deficiency (PSTD) is characterized by apical displacement of the facial mucosal margin and/or deficient peri‐implant soft tissues with exposure of implant or abutment components [5]. Zucchelli et al. classified facial PSTDs at single esthetic‐zone implants according to the facial mucosal margin, bucco‐lingual position of the implant‐supported crown and implant head, and interproximal papillary dimensions [5].

Management is challenging because peri‐implant tissues have reduced vascularity and different connective tissue attachment compared with natural teeth; therefore, flap design and graft handling should preserve vascularity and promote tissue stability [2, 6, 7, 8]. Contemporary guidance also emphasizes prevention and control of peri‐implant disease and maintenance of peri‐implant tissue health [9, 10].

Soft tissue augmentation can improve peri‐implant tissue dimensions, with connective tissue grafting remaining an established approach for increasing tissue thickness [11].

Several techniques have been proposed for PSTDs [12]. Coronally advanced flap with a connective tissue graft (CAF + CTG) is among the most predictable approaches for single implants in the esthetic zone [13], although current evidence remains heterogeneous and additional prospective studies are needed [14].

The laterally closed tunneling (LCT) technique, developed for gingival recession around natural teeth, combines split‐thickness tunnel preparation with controlled lateral mobilization and closure while preserving papillary vascularity [15].

To the best of the author's knowledge, published evidence specifically describing LCT for labial peri‐implant soft tissue dehiscence is limited [12, 14]. A focused PubMed/MEDLINE and Scopus search using terms related to “laterally closed tunnel/tunneling” and “peri‐implant soft tissue dehiscence/deficiency” identified no report specifically describing this application. Accordingly, this report describes the clinical application and biological rationale of LCT combined with a de‐epithelialized free gingival graft for peri‐implant soft tissue augmentation.

## 2 Case Presentation

### 2.1 Patient Information

A 40‐year‐old systemically healthy, non‐smoking male with no history of periodontitis, relevant systemic disease, drug allergy, or medication affecting wound healing, or contributory family or psychosocial history presented to the Department of Oral Medicine and Periodontology, Faculty of Dentistry, The British University in Egypt, complaining of visible metal around an implant replacing the maxillary left central incisor.

Six months earlier, the tooth had been replaced by immediate placement of a 4.0 × 10‐mm Dual Implant System implant (Titan Industries, Cairo, Egypt), a Grade 23 titanium implant with a moderately rough SLA surface, internal conical connection, and platform switching, followed by immediate screw‐retained provisionalization. Because treatment preceded referral, insertion torque, primary stability, insertion depth, abutment/transmucosal details, emergence profile, and detailed restorative records were unavailable. The patient reported gradual facial recession and implant exposure.

**FIGURE 1:**
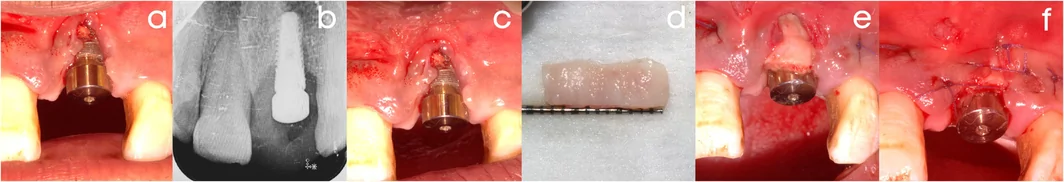
(a) Preoperative clinical view showing marked implant surface exposure at the maxillary left central incisor site. (b) Preoperative standardized periapical radiograph demonstrating the baseline interproximal crestal bone levels adjacent to the implant. (c) Intrasulcular incision and split‐thickness tunnel preparation using the laterally closed tunneling approach. (d) Harvested de‐epithelialized free gingival graft. (e) Placement of the de‐epithelialized free gingival graft within the prepared tunnel. (f) Suturing and lateral closure of the tunnel to achieve tension‐free coverage.

Clinical examination showed a 4 × 3 mm labial PSTD with a thin phenotype (Figure 1a), 1 mm of unattached keratinized mucosa, 1.0‐mm baseline buccal soft tissue thickness, and 2‐mm midfacial probing depth. Papillae were intact and the vestibule was shallow. No bleeding on probing, suppuration, plaque accumulation, mobility, discomfort, or clinical attachment loss at adjacent teeth was detected.

### 2.2 Diagnostic Assessment

#### 2.2.1 Radiographic Assessment

Standardized digital periapical radiographs obtained with the paralleling technique showed the interproximal crestal bone levels adjacent to the implant without evidence of progressive peri‐implant bone loss (Figure 1b). CBCT was not performed because three‐dimensional imaging was not considered clinically justified for this isolated esthetic soft tissue complication; consequently, the three‐dimensional implant position and its relationship to the facial bone could not be assessed.

The defect fulfilled the definition of PSTD according to Zucchelli et al. [5]. A specific Class II–IV subclass was not assigned because the bucco‐lingual position of the implant‐supported crown profile and implant head could not be reliably determined from the available baseline records. The absence of bleeding, suppuration, progressive radiographic bone loss, and other inflammatory signs favored isolated PSTD over peri‐implant mucositis or peri‐implantitis [9, 10].

This case was managed in accordance with the Declaration of Helsinki [16]. According to institutional policy of the Faculty of Dentistry, The British University in Egypt, formal ethical committee approval was not required for a single‐patient case report. Written informed consent was obtained for treatment and for publication of the clinical information and identifiable images.

### 2.3 Therapeutic Intervention

Treatment alternatives included CAF + CTG, prosthetic modification, and clinical monitoring. LCT with a de‐epithelialized free gingival graft was selected to preserve papillary vascularity, avoid vertical releasing incisions, and permit passive lateral tissue mobilization in this shallow‐vestibule esthetic site.

Before surgery, oral hygiene was optimized by scaling, polishing, and oral‐hygiene instruction; 0.12% chlorhexidine (Hexitol, The Arab Drug Company, Cairo, Egypt) was prescribed twice daily for 10 days.

After infiltration with 4% articaine hydrochloride with epinephrine 1:100,000 (Artinibsa 4%, Inibsa Laboratories, Barcelona, Spain), LCT was performed as described by Sculean and Allen [15]. Facial intrasulcular incisions were made with a 15C blade and a split‐thickness tunnel extended beyond the mucogingival junction and beneath adjacent papillae without vertical releases. Sharp and blunt dissection released muscular and collagenous attachments while preserving the periosteal bed and papillary vascularity, permitting passive lateral approximation without perforation (Figure 1c).

Limited implantoplasty was confined to exposed supracrestal threads using a spherical diamond bur in a high‐speed handpiece with continuous copious sterile saline irrigation, consistent with a recently described protocol [17]. Its purpose was to smooth macro‐thread irregularities that might interfere with graft adaptation, not to treat peri‐implantitis. Surrounding tissues were protected, and repeated saline irrigation limited heat and particle accumulation and removed debris before graft placement.

A palatal free gingival graft was harvested as described by Zucchelli et al. [1] and completely de‐epithelialized to obtain an approximately 2‐mm‐thick connective tissue graft matching the tunnel (Figure 1d). It was inserted facing the recipient bed and stabilized mesially and distally with 5–0 Prolene internal fixation sutures; interrupted sutures then approximated the tunnel margins laterally for tension‐free coverage (Figure 1e,f).

The existing screw‐retained provisional restoration was maintained out of occlusal contact and left undisturbed during surgery and healing to avoid mechanical loading of the augmented tissues.

**FIGURE 2:**
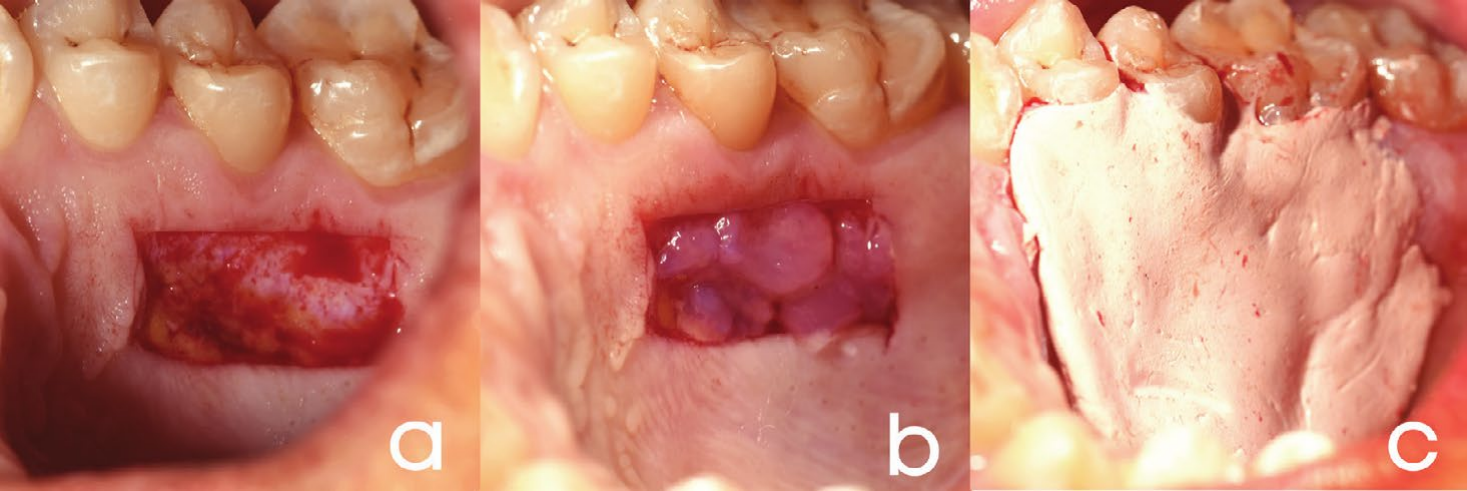
(a) Palatal donor site following graft harvesting. (b) Application of ozonated olive oil gel to the donor site. (c) Placement of a non‐eugenol periodontal dressing.

Following graft harvesting, 0.3–0.5 mL of ozonated olive oil gel (GeliO3; Bioemmei Srl, Vicenza, Italy; 20 mEq O_2_/kg) was applied only to the palatal donor site using a sterile syringe with a blunt 23‐gauge needle, followed by a non‐eugenol periodontal dressing (Pericem, Quengco, Verona, Italy) [18] (Figure 2a–c). It was not part of the peri‐implant intervention.

Postoperative medication comprised amoxicillin 1000 mg orally twice daily for 5 days (Amoxil, GlaxoSmithKline, Cairo, Egypt) and ibuprofen 600 mg twice daily for 5 days (Brufen, Abbott, Cairo, Egypt). A short antibiotic regimen was selected because treatment combined peri‐implant augmentation, palatal harvesting, and implant‐surface modification, consistent with reported connective‐tissue‐grafting implant protocols [19].

The patient continued 0.12% chlorhexidine twice daily for 10 days and avoided brushing or mechanical trauma at the surgical site during early healing [9, 20]. The provisional restoration remained out of occlusion.

### 2.4 Follow‐Up Visits

#### 2.4.1 Two‐Week Follow‐Up (Suture Removal)

**FIGURE 3:**
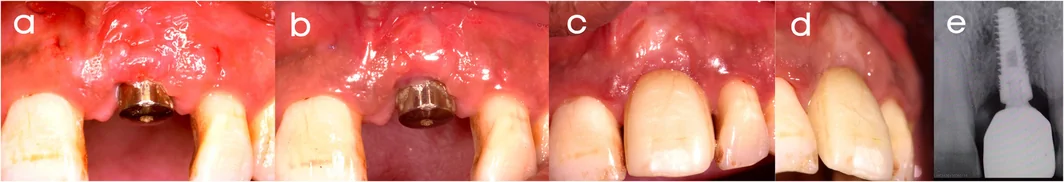
(a) Two‐week postoperative view demonstrating uneventful healing. (b) Three‐month follow‐up showing complete soft tissue coverage and improved esthetic integration. (c) Two‐year follow‐up (labial view) demonstrating stable peri‐implant soft tissue contours. (d) Two‐year follow‐up (lateral view) showing maintained tissue volume and contour. (e) Postoperative periapical radiograph at the two‐year follow‐up demonstrating stable interproximal crestal bone levels.

At 2 weeks, the patient reported adherence to postoperative instructions; healing was uneventful without inflammation, infection, or wound dehiscence; graft integration was satisfactory and sutures were removed (Figure 3a).

#### 2.4.2 Three‐Month Follow‐Up

At 3 months, complete defect coverage was observed, with keratinized mucosa increasing from 1 to 3 mm and buccal soft tissue thickness to 2.4 mm. Esthetic integration improved without biological or technical complications (Figure 3b).

#### 2.4.3 Two‐Year Follow‐Up

At 24 months, complete coverage and peri‐implant tissue contours remained stable around the definitive crown. Buccal soft tissue thickness was 2.7 mm, and standardized periapical radiography showed stable interproximal crestal bone levels (Figure 3c–e).

## 3 Outcomes

**FIGURE 4:**
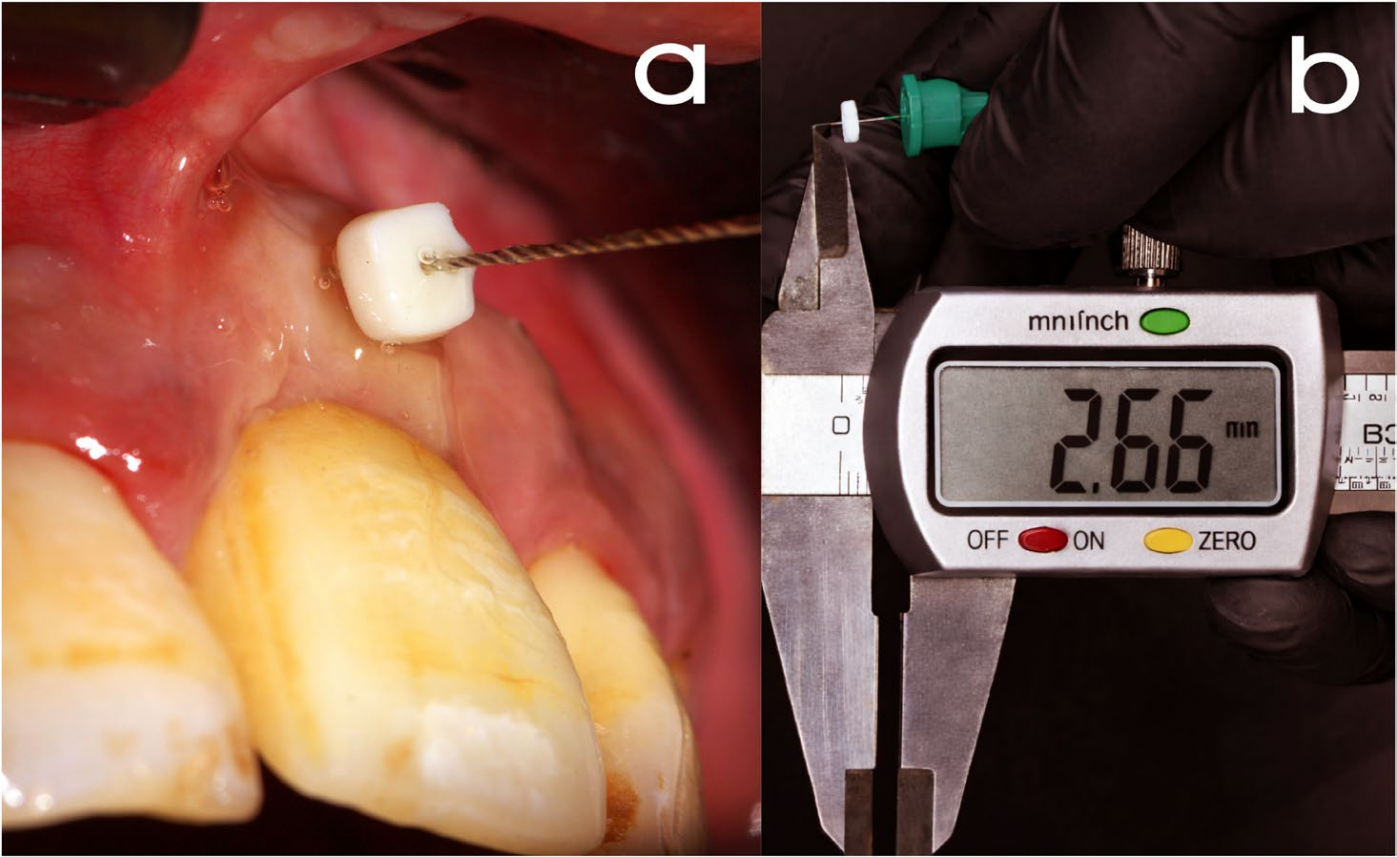
(a) Measurement of buccal soft tissue thickness using an endodontic file with a silicone stopper. (b) Digital caliper used to record the distance between the stopper and the file tip.

Outcomes were evaluated at baseline, 3 months, and 24 months. Midfacial buccal soft tissue thickness was measured by transgingival probing under local anesthesia with an endodontic file and silicone stopper inserted perpendicular to the mucosa until gentle implant‐surface contact; the distance was measured by digital caliper to the nearest 0.1 mm. One measurement was recorded at baseline and 24 months at the same reference (Figure 4). PES was scored from standardized photographs according to Fürhauser et al. [21] by two examiners, with components in Table 1; MSI followed Wessels et al. [22]. Formal examiner agreement/reliability was not assessed and assessors were not blinded to time point. Crestal bone levels were assessed qualitatively on standardized paralleling periapicals [23], and satisfaction on a 0–10 VAS [24].

**TABLE 1:** Individual pink esthetic score (PES) component scores at at baseline, 3‐month, and 24‐month evaluations.

PES variable (Fürhauser et al.)	Baseline	3 months	24 months
Mesial papilla	2	2	2
Distal papilla	2	2	2
Level of facial mucosa	0	2	2
Soft tissue contour	0	1	2
Alveolar process deficiency	0	1	1
Soft tissue color	1	1	2
Soft tissue texture	1	1	1
Total PES	6	10	12

Complete coverage was maintained at 24 months. Buccal soft tissue thickness increased from 1.0 to 2.7 mm, keratinized mucosa from 1 to 3 mm, PES from 6 to 12, MSI from 3 to 0, and satisfaction from 4 to 10; the full outcomes are summarized in Table 2 and the clinical timeline in Table 3.

**TABLE 2:** Clinical, esthetic, radiographic, and patient‐reported outcomes before and after treatment.

Outcome	Baseline	3 months	24 months
Facial peri‐implant soft tissue dehiscence	4 × 3 mm	Complete clinical coverage	Complete coverage maintained
Buccal peri‐implant soft tissue thickness (mm)	1.0	2.4	2.7
Pink Esthetic Score (PES, 0–14)	6	10	12
Mucosal Scarring Index (MSI)	3	1	0
Buccal contour	Pronounced facial concavity	Markedly improved	Harmonious contour (concavity absent)
Keratinized peri‐implant mucosa	1 mm unattached	3 mm (1 unattached, 2 mm attatched)	Improvement maintained
Radiographic assessment (standardized periapical radiographs)	Baseline interproximal crestal bone level	Stable	Stable interproximal crestal bone level without radiographic evidence of progressive bone loss
Patient satisfaction (VAS, 0–10)	4	9	10

**TABLE 3:** Timeline of the clinical course of the case.

Time point	Clinical event
6 months before presentation	Immediate implant placement and provisional restoration following extraction of the hopeless maxillary left central incisor.
Approximately 3 months after implant placement	Patient noticed gradual labial soft tissue recession with increasing metal exposure.
Baseline examination	Patient presented with an esthetic complaint related to visible implant threads. Comprehensive clinical and radiographic examination performed. Diagnosis established.
Pre‐surgical preparation	Oral hygiene optimization, scaling and polishing, chlorhexidine mouth rinse for 10 days, and Treatment planning completed.
Day of surgery	Implantoplasty, Laterally closed tunneling (LCT) with de epithelized free gingival graft placement and stabilization.
Immediate postoperative period	Antibiotics, analgesics, and chlorhexidine mouth rinse prescribed; provisional restoration kept out of occlusion
2 weeks follow‐up	Uneventful healing; graft integration confirmed; sutures removed
3 months follow‐up	Complete soft tissue coverage achieved; increased tissue thickness and improved esthetic outcome
6 months follow‐up	Final prosthetic rehabilitation; Definitive crown delivered
24 months follow‐up	Stable peri‐implant soft tissue conditions; successful final restoration; no recurrence or complications

## 4 Discussion

Peri‐implant soft tissue dehiscence remains challenging because vascularity and biologic resilience are limited compared with periodontal tissues [1, 2, 6]. In this case, LCT was chosen to preserve papillary integrity and permit tension‐free lateral approximation without vertical releases, features potentially advantageous when coronal advancement may be constrained by vestibular depth or muscular pull [13, 15].

The observed coverage may reflect the combination of passive tunnel mobilization, volumetric augmentation with a de‐epithelialized free gingival graft, and preservation of papillary vascularity [1, 11, 15]. At 24 months, coverage, increased tissue thickness, esthetic scores, and patient satisfaction remained stable. These findings are consistent with biologically driven peri‐implant soft tissue augmentation concepts, but a single case cannot establish comparative efficacy or predictability [11, 14].

Limited implantoplasty represented a modification of the original LCT technique. Unlike implantoplasty for peri‐implantitis, it was used here solely to smooth exposed supracrestal macro‐threads and facilitate soft tissue adaptation, not to treat infection [17, 25]. A similar combination with connective tissue grafting has been described by Neto et al. [17]. Because implantoplasty is irreversible and may reduce implant wall thickness and fracture resistance [26], surface modification was restricted to exposed threads and performed with copious irrigation and subsequent saline cleansing.

Strengths of this report include the 24‐month follow‐up and assessment of clinical, esthetic, radiographic, and patient‐reported outcomes. Limitations include the single‐case design and unavailable pre‐referral data on insertion torque, primary stability, insertion depth, abutment characteristics, and precise three‐dimensional implant position. Transgingival probing has biological and operator variability; one measurement was recorded, formal examiner reliability was not assessed, and assessors were not blinded to time point. Because CBCT was not clinically indicated, facial bone and three‐dimensional implant position could not be evaluated; standardized periapicals allowed only qualitative assessment of interproximal crestal bone levels. Controlled studies are required to establish indications, reproducibility, and long‐term outcomes.

## 5 Conclusions

In this single case, LCT combined with a de‐epithelialized free gingival graft permitted tension‐free closure with preservation of the papillae and was associated with maintained coverage, increased soft tissue thickness, and favorable esthetic outcomes at 24 months. Further controlled studies are required before the predictability of this approach around implants can be established.

### 5.1 Patient Perspective

The patient reported high satisfaction with the esthetic result. Before surgery, implant exposure had affected his confidence when smiling; recovery was uneventful, and he remained satisfied with the improved soft tissue appearance and stated that he would choose the same treatment again.

## Author Contributions

**Hisham Tarek:** writing – original draft, conceptualization, investigation, funding acquisition, methodology, validation, visualization, writing – review and editing, project administration, formal analysis.

## Funding

The author has nothing to report.

## Ethics Statement

According to institutional policy of the Faculty of Dentistry, The British University in Egypt, formal ethical approval was not required for this single‐patient case report. The patient was informed of the irreversible nature, potential risks and benefits, and alternatives to implant surface modification and provided written informed consent before treatment.

## Consent

This case report was conducted in accordance with the Declaration of Helsinki. Written informed consent was obtained for treatment and, separately, for publication of the clinical information and accompanying clinical photographs.

## Conflicts of Interest

The author declares no conflicts of interest.

## Data Availability

The data that support the findings of this study are available from the corresponding author upon reasonable request.
